# *Mycobacterium tuberculosis* PE_PGRS38 Enhances Intracellular Survival of Mycobacteria by Inhibiting TLR4/NF-κB-Dependent Inflammation and Apoptosis of the Host

**DOI:** 10.3390/biology13050313

**Published:** 2024-04-30

**Authors:** Hayan Ullah, Xiaoxia Shi, Ayaz Taj, Lin Cheng, Qiulong Yan, Shanshan Sha, Ahmad, Jian Kang, Muhammad Haris, Xiaochi Ma, Yufang Ma

**Affiliations:** 1 Department of Biochemistry and Molecular Biology, Dalian Medical University, Dalian 116044, China; hayan.khan12@gmail.com (H.U.); mayaztaj@hotmail.com (A.T.); cl941515685@163.com (L.C.); shanshan_sha@dmu.edu.cn (S.S.); kangjian_dmu@dmu.edu.cn (J.K.); mharismic@yahoo.com (M.H.); 2 Department of Microbiology, Dalian Medical University, Dalian 116044, China; qiulongyan@dmu.edu.cn; 3 Department of Experimental Teaching Center of Public Health, Dalian Medical University, Dalian 116044, China; shixx01@dmu.edu.cn; 4 Department of Immunology, Dalian Medical University, Dalian 116044, China; 2720100102004@dmu.edu.cn; 5 Pharmaceutical Research Center, The Second Affiliated Hospital, Dalian Medical University, Dalian 116044, China

**Keywords:** *Mycobacterium tuberculosis*, PE_PGRS family, PE_PGRS38, *Mycobacterium smegmatis*, virulence factor

## Abstract

**Simple Summary:**

PE_PGRS38 is a member of the PE/PPE protein family of *M. tuberculosis*. PE_PGRS members are unique, restricted to virulent mycobacteria, and their role in host–pathogen interactions is not yet fully elucidated. In this study, we constructed recombinant strains of *M. smegmatis* expressing the PE_PGRS38 protein of *M. tuberculosis*. Using in vitro and in vivo infection models, we investigated the role of PE_PGRS38 in host–pathogen interactions and the pathogenicity of tuberculosis. PE_PGRS38 enhanced the intracellular survival of recombinant *M. smegmatis* by downregulating proinflammatory responses. PE_PGRS38 also inhibited inflammation, the inflammasome, and apoptosis in RAW264.7 cells during infection. Similarly, PE_PGRS38 inhibited the expression level of TLR4 and NF-ĸB in the lungs of infected mice. These findings indicate that PE_PGRS38 is a potential virulence factor that contributes to pathogenicity by evading the host’s immune responses and could aid in the development of antimycobacterial drugs and vaccines.

**Abstract:**

*Mycobacterium tuberculosis* (Mtb) ranks as the most lethal human pathogen, able to fend off repeated attacks by the immune system or medications. PE_PGRS proteins are hallmarks of the pathogenicity of Mtb and contribute to its antigenic diversity, virulence, and persistence during infection. *M. smegmatis* is a nonpathogenic mycobacterium that naturally lacks PE_PGRS and is used as a model to express Mtb proteins. PE_PGRS has the capability to evade host immune responses and enhance the intracellular survival of *M. smegmatis*. Despite the intense investigations into PE_PGRS proteins, their role in tuberculosis remains elusive. We engineered the recombinant *M. smegmatis* strain Ms-PE_PGRS38. The result shows that PE_PGRS38 is expressed in the cell wall of *M. smegmatis*. PE_PGRS38 contributes to biofilm formation, confers permeability to the cell wall, and shows variable responses to exogenous stresses. PE_PGRS38 downregulated TLR4/NF-κB signaling in RAW264.7 macrophages and lung tissues of infected mice. In addition, PE_PGRS38 decreased NLRP3-dependent IL-1β release and limited pathogen-mediated inflammasome activity during infection. Moreover, PE_PGRS38 inhibited the apoptosis of RAW264.7 cells by downregulating the expression of apoptotic markers including Bax, cytochrome c, caspase-3, and caspase-9. In a nutshell, our findings demonstrate that PE_PGRS38 is a virulence factor for Mtb that enables recombinant *M. smegmatis* to survive by resisting and evading the host’s immune responses during infection.

## 1. Introduction

*Mycobacterium tuberculosis* (Mtb) causes tuberculosis (TB), an infectious illness that may spread from person to person and often persists throughout a person’s life [1]. According to World Health Organization Global Tuberculosis Report, 10.6 million people have been diagnosed with TB and 1.6 million of them died, including 187,000 HIV-positive individuals. Although TB is prevalent worldwide, low-income countries account for the majority of cases. Half of the TB cases are in Bangladesh, India, China, Nigeria, Indonesia, Pakistan, South Africa, and the Philippines [2]. Mtb’s genome encodes for unique proline-glutamic acid (PE) and proline-proline-glutamic acid (PPE) family proteins that occupy 10% of the genome [3]. The nomenclature assigned to the protein family is PE and PPE, based on the existence of distinct amino acid motifs, proline-proline-glutamic acid (PPE), and proline-glutamic acid (PE) located in the N-terminal region. Cell-wall-associated multiple-gene families coding PE and PPE families were identified via interpreting the entire genome of *Mycobacterium tuberculosis* H37Rv [4,5]. The PE family exhibits a dichotomy, comprising two distinct subfamilies, such as PE and PE_PGRS. The PE_PGRS subfamily consists of N-terminal PE and C-terminal PGRS (polymorphic GC-rich repetitive sequences). The C-terminal PGRS region consists of a polymorphic domain that contains Gly-Gly-Ala and Gly-Gly-Asn amino acid repeats. These mycobacterial proteins are located in the matrix of the outer membrane and contribute to the pathogenesis of Mtb [6].

Alveolar macrophages and dendritic cells (DC) play a major role in the first-line innate immune system. The initial and most crucial stage in Mtb pathogenesis is the adhesion of the pathogen to the alveolar macrophages and dendritic cells (DC). To execute the eradication of pathogens, macrophages are required to recognize, phagocytize, and eliminate Mtb. Macrophages execute infiltration of adaptive immune responses by secretion of proinflammatory cytokines, chemokines, and antigens through MHCI and MHCII during infection [7,8,9]. However, Mtb strategically evades the host antimycobacterial responses and enhances its intracellular survival by hijacking the fusion of the phagosome with the lysosome [10].

The precise functions of PE_PGRS are not yet elucidated. However, little is known about their role in Mtb virulence, particularly during the chronicity and latency stages [11]. Various studies have reported that PE_PGRS family proteins have FnBP-Fn-binding properties that contribute to the attachment of bacteria to host receptors and invade host cells [12,13,14,15]. Due to the unique phenotypic architecture of the PE_PGRS family, these proteins are crucial to provoking innate immune responses, including inflammation [16], apoptosis [17], and adaptive immune responses [16]. Several members of the PE_PGRS family are responsible for mycobacterial abundance inside the cell [18,19,20]. Recent studies have investigated the potential role of PE_PGRS members in the downregulation of proinflammatory responses [21,22], apoptosis, and autophagy [23]. Another study explored the efficacy of a DNA vaccine utilizing the Rv1818c protein of Mtb to evoke immune responses in mice. The vaccine elicited potent cellular immunity, marked by IFN-gamma-producing CD8+ T cells that are capable of eliminating antigen-presenting cells. Additionally, two peptides from Rv1818c were identified as epitopes with strong binding affinity to MHC molecules, prompting the activation of CD8+ T cells [24,25]. Mutations in *M. marinum* homologs of Rv3812 and Rv1651c decreased granuloma persistence [26]. These investigations highlighted the significance of PE_PGRS family members in the pathogenesis of tuberculosis.

PE_PGRS38 is a key member of the PE_PGRS protein family, located in the Mtb complex-specialized genomic island. A previous study reported the expression and interaction of PE_PGRS38 with HAUSP that caused the inhibition of deubiquitination of tumor necrosis receptor associated receptor 6 (TRAF6)-dependent inflammatory responses in bone-marrow-derived macrophages (BMDM) and 293T cell lines. The role of PE_PGRS38 in the enhanced intracellular survival of bone-marrow-derived macrophages (BMDM), 293T cell lines, and in the lungs of infected mice has also been reported in a previous study [27]. However, the localization and impacts of PE_PGRS38 on cell wall integrity and biofilm formation have not yet been explored. Furthermore, the role of PE_PGRS38 in TLR4/NF-ĸB-dependent inflammation, the inflammasome, and apoptosis during infection has remained elusive. As *M. smegmatis* lacks PE_PGRS family proteins, we designed and generated a recombinant *M. smegmatis* expressing PE_PGRS38, namely Ms-PE_PGRS38, to uncover its role in virulence. We detected the expression, localization, stress responses, and biofilm potential of PE_PGRS38 in *M. smegmatis*. We also demonstrated the contribution of PE_PGRS38 in the pathogenesis of tuberculosis by investigating its role in intracellular survival, inflammation, the inflammasome, and apoptosis of the infected RAW264.7 macrophages. We also studied its pathogenic capability and found that PE_PGRS38 increased the burden of recombinant mycobacteria and inhibited the expression level of TLR4/NF-ĸB in the lungs of C57BL/6J mice. PE_PGRS38 also downregulated the inflammation in the lungs of infected mice. Together, our results show that PE_PGRS38 is a potential virulence factor that plays a pivotal role in the pathogenesis of tuberculosis.

## 2. Materials and Methods

### 2.1. Bacterial Strains and Plasmids

The NovaBlue strain of *Escherichia coli* (*E. coli*) was obtained from Novagen (Madison, WI, USA) and grown on LB agar and broth. *M. smegmatis* mc^2^155 (ATCC, Manassas, VA, USA) was cultured on Middlebrook 7H9 broth and Middlebrook 7H11 agar, enriched with 0.05% Tween 80, 10% albumin-dextrose-catalase (ADC), and 0.5% glycerol. The pJET1.2/blunt vector was purchased from Thermo Fisher Scientific and used to clone the PE_PGRS38 gene. The *E. coli* NovaBlue was used to propagate the recombinant pJET-PE_PGRS38 gene. The pVV2 plasmid carrying the BCG hsp60 promotor and N-terminal His-tag was obtained from Colorado State University, USA. Ampicillin (Amp) and kanamycin (Kan) were bought from Sigma (Kanagawa, Japan) and employed at the defined concentrations: Amp, 100, and Kan, 50 µg/µL for *E. coli* and 25 µg/µL Kan for *M. smegmatis* mc^2^155.

### 2.2. Cell Line and Mice

RAW264.7 macrophage cells were grown in Dulbecco Modified Eagle Medium (DMEM), supplemented with 10% fetal bovine serum (FBS) (Lonsera, Canelones, Uruguay), 100 U mL^−1^ penicillin, and 100 µg mL^−1^ streptomycin. Specific pathogen free (SPF) female C57BL/6J mice, 8 weeks old, were kept at the SPF animal facility at the Laboratory Animal Center of Dalian Medical University, China. After infection by bacteria, the mice were maintained in a clean environment illuminated for 12 h each day and kept at 24 ± 1 °C with free access to sterilized food and water. Animal experiments adhered to the NIH guide for the care and use of laboratory mice and received approval from the Dalian Medical University Committee on the Ethics of Animal Experiments [28]. All experiments on mice were designed to minimize the number of subjects used, and distress and suffering were minimized as much as possible for the animals.

### 2.3. Construction of Recombinant M. smegmatis Strains

PE_PGRS38 was amplified through PCR from the genomic DNA of *M. tuberculosis* H37Rv. Primers are listed in the Supplementary Table S1. The underlined CATATG and GGATCC depict the restriction recognition site sequences for NdeI and BamHI. The recombinant pJET-PE_PGRS38 plasmid was made by cloning the PCR product of PE_PGRS38 from H37Rv into the cloning vector pJET1.2/blunt. After approval by DNA sequencing, the PE_PGRS38 gene was digested from the pJET-PE_PGRS38 plasmid by NdeI (TaKaRa, Kyoto, Japan) and BamHI (TaKaRa, Kyoto, Japan). The digested PE_PGRS38 was then ligated into the pVV2 (expression vector) to construct the pVV2-PE_PGRS38 plasmid. The pVV2-PE_PGRS38 and empty pVV2 vectors were electroporated into *M. smegmatis* mc^2^155 cells [29] to generate the recombinant *M. smegmatis* Ms-pVV2 and Ms-PE_PGRS38 strains.

### 2.4. Detection of Recombinant PE_PGRS 38 in M. smegmatis

The Ms-pVV2 and Ms-PE_PGRS38 recombinant strains were grown in 7H9 until the OD_600_ value (turbidity) reached 1. The cultures of Ms-pVV2 and Ms-PE_PGRS38 strains were then harvested via centrifugation at 3200× *g* for 10 min at 4 °C. The cells were then sonicated in cold lysis buffer containing protease inhibitor phenylmethanesulfonyl fluoride (PMSF) added to it (Sigma-Aldrich, Saint Louis, MO, USA). The sonicated whole-cell lysates were centrifuged at 3200× *g* to separate normal bacteria (pellets) and whole-cell lysate fractions (WCL). Equal quantities of WCL from the Ms-pVV2 and Ms-PE_PGRS38 strains were processed through sodium dodecyl sulfate-polyacrylamide gel electrophoresis (SDS-PAGE), followed by electrotransfer to a nitrocellulose membrane. The membrane was then incubated with anti-polyhistidine clone HIS-1 antibody (Sigma-Aldrich) overnight at 4 °C, followed by HRP-conjugated secondary antibody (Proteintech, Rosemont, IL, USA), and visualized via ECL solution.

### 2.5. Localization of Recombinant PE_PGRS38 Protein in M. smegmatis

Cultures of Ms-pVV2 and Ms-PE_PGRS38 were harvested and lysed through sonication. To separate the whole bacteria, the WCLs of Ms-pVV2 and Ms-PE_PGRS38 were centrifuged at 3200× *g* for 10 min at 4 °C. Subsequently, the supernatant fractions were centrifuged at 27,000× *g* for 30 min at 4 °C. The pellets (cell wall, CW) and the supernatants (cytoplasm, CP) were collected. Both pellet and supernatant fractions were subjected to Western blotting. Blots were visualized via the Chemi-doc system (BioRad, Hercules, CA, USA) to detect the expression of and localization of PE-PGRS38. GroL2 (cytoplasmic marker protein) was used as a control.

### 2.6. Biofilm Assay

For the evaluation of biofilm formation, we used the protocol of the previous study with a few modifications [30]. Ms-pVV2 and Ms-PE_PGRS38 were maintained in M63 broth at 37 °C. When OD600 reached 0.5, cultures were diluted 1:1000 in M63 growth medium with 0.7 mM CaCl_2_, 1 mM MgSO_4_, and 0.5% casamino acids. Each 96-well polystyrene microtiter plate well was filled with 150 µL of culture. In static conditions, recombinant strains were incubated for 5 days at 30 °C. Three times, the wells were rinsed with physiological saline, and for staining, 150 µL of 1% crystal violet was added. After 15 min of incubation, the plates were washed three times. Bacterial cells were washed with 95% ethanol, and biofilm formation was measured through optical density at 570 nm (OD_570_).

### 2.7. Growth and Survival of Recombinant M. smegmatis under Different In Vitro Stresses

To inspect the growth patterns of Ms-pVV2 and Ms-PE_PGRS38, the bacteria were grown to an OD_600_ of 0.8. The OD_600_ value was taken at a 12 h interval. For stress assessment, Ms-pVV2 and Ms-PE_PGRS38 were challenged with H_2_O_2_, acidic pH, and sodium dodecyl sulfate stress. For treatment with H_2_O_2_, recombinant strains were supplemented with 10 mM H_2_O_2_ for 1, 2, 3, and 4 h, respectively. For acidic stress, pH was adjusted to 3 by supplementing HCl to 7H9 broth. The 7H9 broth was then filter sterilized by a 0.22 μm filter, and recombinant strains were exposed to acidic pH for 3, 6, and 9 h. For SDS stress, both recombinant strains were exposed to 0.05% SDS for 1, 2, 3, and 4 h. After the exposure to H_2_O_2_, acidic stress, and SDS, the recombinant cultures were serially diluted at 1:10 and spotted onto 7H11 agar plates, and CFU were counted after 60–72 h.

### 2.8. In Vitro and In Vivo Survival Measurement of Recombinant M. smegmatis

RAW264.7 cells were seeded in 24-well dishes with 0.5 × 10^5^ cells/well density for 24 h. Before infection, recombinant *M. smegmatis* was collected at 3200× *g* for 5 min and suspended in DMEM medium without antibiotics or FBS. RAW264.7 cells were washed with PBS and infected with recombinant *M. smegmatis* strains (Ms-pVV2 and Ms-PE_PGRS38). The cells were infected at a 10:1 multiplicity of infection (MOI) for 4 h [31,32]. The RAW264.7 cells were washed with PBS to eliminate extracellular recombinant *M. smegmatis* and then maintained in DMEM supplemented with 10% FBS. This time point was considered as 0 h. The cells were lysed by adding 0.03% *w*/*v* SDS for 3–5 min, and colony-forming units (CFU) at 0, 6, 12, 18, and 24 h post-infection were performed. The RAW264.7 lysates were 10-fold serially diluted and cultured on 7H11 agar, and the colonies were calculated 60–72 h post-inoculation. For in vivo, we administered recombinant *M. smegmatis* to C57BL/6J mice using aerosol-producing equipment (Kangjie Instrument, Taizhou, China). Aerosolized *M. smegmatis* in PBST was given to each mouse daily at 5 × 10^9^ CFU for 4 days [33]. Three mice from each group were euthanized and sacrificed at 4, 8, and 16 days post-infection. The entire right lobe of each mouse’s lung was aseptically homogenized in PBS. The resultant homogenates were diluted and seeded on 7H11 agar. Bacterial burden in the lung was determined by CFU after 60–72 h of incubation at 37 °C.

### 2.9. Real-Time qPCR (RT-qPCR) Assay for mRNA of Cytokines

RAW264.7 at a density of 1 × 10^6^ cells/well was added in 6 well dishes for 24 h before infection. Cells were challenged with 0.1 µg/mL lipopolysaccharides (LPS) (Seven Biotech, Beijing, China) 2 h prior to infection. The cells were then infected with recombinant Ms-pVV2 and Ms-PE_PGRS38 strains at MOI 10 for 4 h and incubated for 24 h post-infection. The total RNA of cells was extracted via RNAiso Plus reagent (Takara, Osaka, Japan) following the manufacturer’s protocol. To make cDNA, reverse transcription of 1 µg/µL RNA was performed with the PrimeScript RT Reagent Kit with genomic DNA Eraser (Takara, Japan). An equal quantity of template (cDNA) was processed through RT-qPCR. The reaction was carried out in StepOnePlus Real-Time PCR (Applied Biosystems, San Francisco, CA, USA) using SYBR Green Premix Ex Taq II (Takara) and gene-specific primers (Supplementary Table S1). Conditions for amplification were as follows: 30 s for initial denaturation at 95 °C, 5 s at 95 °C, and 30 s at 60 °C for 40 cycles. The relative expression of specific genes in three independent experiments was evaluated via the 2^−∆∆Ct^ method.

### 2.10. Western Blot

RAW264.7 macrophages were infected with Ms-pVV2 and Ms-PE_PGRS38 strains, as discussed previously. Macrophages were washed with cold PBS three times and then lysed in lysis buffer (Solar Bio, Beijing, China) and 1 mM PMSF. Lysate suspensions were spun at 12,000× *g* for 20 min, and the protein quality was determined using bicinchoninic acid (BCA) (TIANGEN Biotechnology, Beijing, China). An equal quantity of proteins was loaded into the wells, separated by SDS-PAGE, and then electrotransferred to nitrocellulose membranes. The membrane was blocked with 7% non-fat milk and kept for 2 h at room temperature with gentle shaking. Respective primary antibodies for inflammation included TLR4, MYD88, NF-κB (Proteintech, Rosemont, IL, USA), p-NF-κB, IKB-α, and p-IKB-α (Bios, Hong Kong, China). For apoptosis, Bcl-2, Bax, caspase-3, cytochrome c, and caspase-9 (kindly from Abmart, Shanghai, China) were used. For NLRP3-dependent inflammasomes, NLRP3, caspase-1, and IL-1β (Proteintech, USA) were used, while β-actin (Proteintech, USA) served as an internal control. After overnight incubation with the primary antibody, membranes were kept at room temperature for 1 h with corresponding HRP-coupled anti-antibodies (Proteintech, USA). Then, Plus-ECL chemiluminescent reagent (Advansta, San Jose, CA, USA) was used to project the blots.

### 2.11. Apoptosis Assay of Infected RAW264.7 Macrophage Cells

Apoptotic RAW26.7 macrophage cells were determined using the apoptosis detection kit (CWBIO, Taizhou, China). RAW264.7 cells were seeded at 10^6^/mL density and infected with Ms-pVV2 and Ms-PE_PGRS38 strains at 10:1 MOI for 24 h. Then, macrophages were washed with PBS. Cells were digested with trypsin and centrifuged at 900 rpm for 3 min. Cells were washed twice in 1 mL of ice-cold PBS and centrifuged at 900 rpm for 3 min at room temperature. Next, each sample of cells was resuspended in 250 µL of fixing buffer. Then, 100 µL from each sample was shifted to a new tube, and 10 µL of propidium iodide (PI) and 5 µL of Annexin V-FITC were added and kept at room temperature in the dark for 15 min. Macrophages were subjected to flow cytometry (Mindray, Shenzhen, China) and inverted fluorescent microscopy (Olympus, Tokyo, Japan) analysis. Apoptosis of macrophages was measured according to manufacturer protocol by attachment of Annexin V-FITC to the cells. Flowjo 10.8.1 software was used to analyze Annexin V-FITC and PI.

### 2.12. Histopathology Analyses

Each mouse’s upper left lung lobe was excised and stored in 4% PFA (Seven, Xiangtan, China) at 4 °C for 24 h, followed by drying, embedding in paraffin, and sectioning at 3 mm on a rotary microtome (ThermoFisher Scientific, Waltham, MA, USA). A tissue section of each mouse was stained with hematoxylin and eosin (H&E). The pathomorphological changes of the lung sections were detected using a Nikon Ni-U microscope (Nikon, Tokyo, Japan).

### 2.13. Immunofluorescence Assay

Dewaxed lung tissue sections were rehydrated before antigen heat retrieval in citrate buffer, followed by 25–30 min of blocking with 10% goat serum at 25 °C. The samples were then incubated overnight at 4 °C with 1:100 dilutions of murine antibodies against TLR4 (Proteintech, USA) and p-NF-ĸB (Bioss, Beijing, China). Sections were stained for 1 h at room temperature with anti-mouse lgG (H + L) and HRP-fused fluorescent secondary antibody (RRID = AB_2264785) at a 1:100 dilution (Proteintech, USA). The sections were subsequently washed three times with PBS and stained for 10 min at room temperature with DAPI (Seven, China). The images were obtained using an inverted fluorescence microscope (Olympus, Japan).

### 2.14. Statistical Analysis

All statistical analyses were performed using GraphPad Prism 9.2.0 (GraphPad Software, Inc., San Diego, CA, USA). For comparison between the two groups, a *t*-test was employed, while one-way ANOVA (Tukey’s multiple comparisons test) was used for comparing more than two groups.

## 3. Results

### 3.1. Generation of Ms-PE_PGRS38 Recombinant Strain

To generate the recombinant Ms-PE_PGRS38 strain and express PE_PGRS38 protein in *M. smegmatis*, the mycobacterial expression vector pVV2 and PE_PGRS38 were ligated (Figure S1A–C). Furthermore, localization was performed through fractionation followed by Western blot, and the results show that PE_PGRS38 expressed in the cell wall fraction (Figure S1D). Overexpression of PE_PGRS38 has no effect on the growth of mycobacteria (Figure S1E).

**Figure 1:**
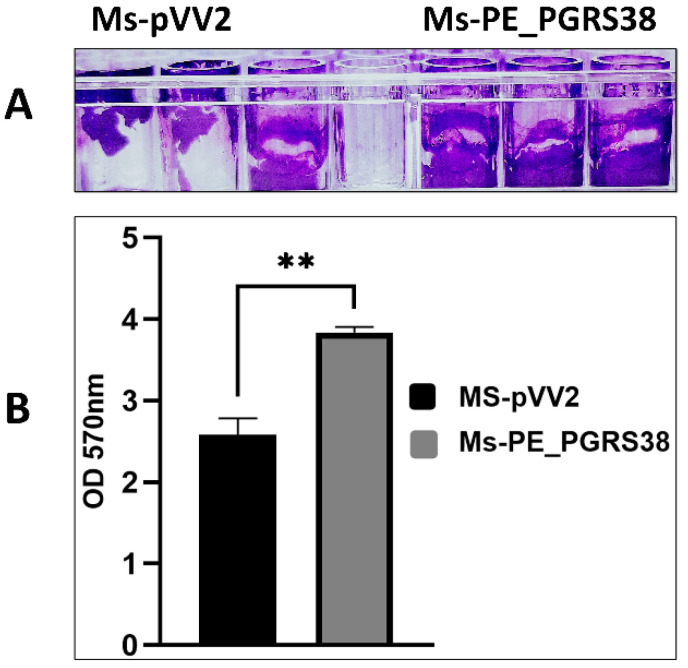
PE_PGRS38 promoted biofilm formation in *M. smegmatis* mc^2^155. Ms-pVV2 and Ms-PE_PGRS38 were maintained under the same growth and culturing conditions. (**A**) represents the quantitative analysis of biofilm formation ability via crystal violet, and (**B**) indicates the densitometric analysis of biofilm formation. The bar graph represents the densitometric analysis of biofilm for each group of bacteria. ** *p* < 0.001 (unpaired *t*-test); the data represent the experiments with three independent biological replicates.

Biofilm is an organized bacterial community covered in self-secreted extracellular polymeric substrates (EPSs). Compared to free-living planktonic cells, bacteria in biofilms have greater chances of survival under adverse conditions such as nutrient shortage, dehydration, pH fluctuations, and antimicrobial agents [34,35]. Being part of the cell wall, we aimed to inspect the role of the PE_PGRS38 protein in biofilm formation in *M. smegmatis.* Under the same growth and culturing conditions, biofilm formation in Ms-PE_PGRS38 was significantly increased as compared to Ms-pVV2 (Figure 1).

### 3.2. Response of Engineered M. smegmatis Strains to In Vitro Stresses

**Figure 2:**
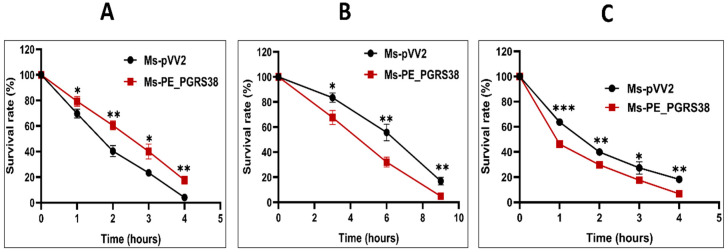
PE_PGRS38 changed the permeability of the cell wall of recombinant *M. smegmatis.* (**A**) Mid-log-phase Ms-pVV2 and Ms-PE_PGRS38 were cultured in 7H9 supplemented with 10 Mm H_2_O_2_ for indicated times_._ (**B**) Mid-log-phase Ms-pVV2 and Ms-PE_PGRS38 were collected by centrifuge and suspended in acidic 7H9 with a pH of 3.0 for the indicated times. (**C**) Mid-log-phase Ms-pVV2 and Ms-PE_PGRS38 were inoculated in 7H9 supplemented with 0.75% SDS and incubated for specific intervals of time. Ten-fold serial dilutions of recombinant strains were inoculated on 7H11 agar augmented with 10% ADC and 25 µg/mL kanamycin. Colonies of Ms-pVV2 and Ms-PE_PGRS38 were calculated after 60–72 h. * *p* < 0.05, ** *p* < 0.01 and *** *p* < 0.001 (*t*-test).

The host cell naturally exposes the phagocytized microbe in the phagosome to stress environments, such as low pH and reactive oxygen species, as an antimicrobial strategy for pathogen elimination [36]. To find out how recombinant strains react, we grew Ms-pVV2 and Ms-PE_PGRS38 in 7H9 media under conditions that mimic the stress of the host, like 10 mM H_2_O_2_ and a pH of 3.0. Ms-pVV2 and Ms-PE_PGRS38 strains were cultured in 7H9 media supplemented with 10 mM H_2_O_2_, and survival was assessed at four time points. Overexpression of PE_PGRS38 promoted the viability of Ms-PE_PGRS38 under H_2_O_2_ stress as compared to Ms-pVV2 (Figure 2A). Resistance of Ms-PE_PGRS38 to H_2_O_2_ could contribute to the intracellular survival of mycobacteria during infection.

Limited acidification in the phagosome environment is one of the hallmarks of the host alveolar macrophages during Mtb infection [36]. To figure out the role of PE_PGRS38 in stress tolerance, recombinant Ms-pVV2 and Ms-PE_PGRS38 were incubated in acidic 7H9, and the survival rates of recombinant strains were measured at different times at pH 3.0.

The survival of Ms-PE_PGRS38 at pH 3.0 was drastically decreased as compared to Ms-pVV2, which indicated that the overexpression of PE_PGRS38 changed the integrity of the cell wall of *M. smegmatis* (Figure 2B). Ms-pVV2 and Ms-PE_PGRS38 were cultured in 7H9 media supplemented with 0.75% sodium dodecyl sulfate. The susceptibility of recombinant strains was observed at four time points, which shows that Ms-PE_PGRS38 is highly vulnerable to 0.75% SDS as compared to Ms-pVV2 (Figure 2C). Together, stress results demonstrate that PE_PGRS38 has impacts on cell wall architecture and the integrity of the cell.

### 3.3. Persistence of Ms-PE_PGRS38 In Vitro and In Vivo

Several PE_PGRS family proteins are vital for the virulence and persistence of Mtb in macrophages [31,32,33]. Previous investigations have revealed that PE_PGRS proteins promote mycobacteria’s survival within macrophages [37,38,39]. Kim et al. reported that PE_PGRS38 enhanced the intracellular survival of *M. smegmatis* in BMDM and 293T cell lines and in the lungs of infected mice [27]. To examine PE_PGRS38′s role in the virulence of mycobacterium in vitro and in vivo, we performed an infection of RAW264.7 and C57BL/6J mice with Ms-pVV2 and Ms-PE_PGRS38 and compared their survival. In line with the previous study, our results show that PE_PGRS38 increased the intracellular survival of Ms-PE_PGRS38 as compared to Ms-pVV2 (Figure S2A,B). These findings demonstrate that PE_PGRS38 is a virulence factor and promotes the intracellular survival of *M. smegmatis*.

### 3.4. PE_PGRS38 Disturbed Regulation of Proinflammatory Cytokines in RAW264.7 Macrophages

**Figure 3:**
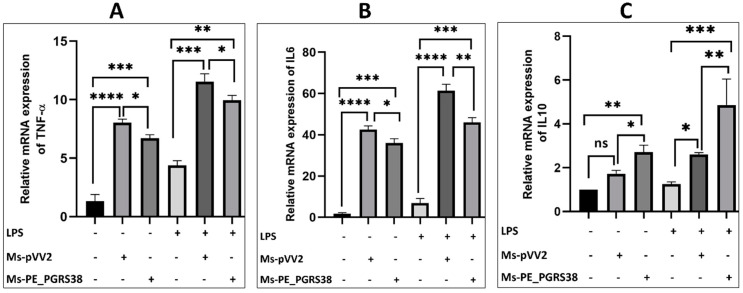
PE_PGRS38 modulated the expression of proinflammatory markers. RAW264.7 macrophage cells were seeded for 24 h and challenged with LPS 2 h prior to infection, respectively. (**A**) represents TNF-α, (**B**) IL-6, and (**C**) IL-10. ns > 0.05, * *p* < 0.05 and ** *p* < 0.01, *** *p* < 0.001, **** *p* < 0.0001 (one-way ANOVA).

Pro-inflammatory cytokines are the critical immune responses of macrophages controlled by NF-κB and MAP-K signaling pathways in response to external stimuli. Virulence factors in Mtb have the ability to induce inflammation. Kim et al. investigated that PE_PGRS38 downregulates the proinflammatory cytokines in BMDM and 293T cell lines [27]. We used Ms-pVV2 and Ms-PE_PGRS38 for infection of RAW264.7 macrophages to study their effects on TNF-α, IL-1β, IL-6, and IL-10 mRNA expression. Our results show that Ms-PE_PGRS38 inhibited the expression of proinflammatory signals while upregulating IL-10 expression as compared to Ms-pVV2 (Figure S3A–D). To validate the cytokine inhibition efficacy of PE_PGRS38, we treated RAW264.7 cell lines with 0.1 µg/mL LPS 2 h prior to infection with mycobacteria. Similarly, Ms-PE_PGRS38 inhibited the expression level of proinflammatory cytokines in LPS-primed RAW264.7 as compared to Ms-pVV2 (Figure 3), (Figure 5C). Moreover, the immunoblot results show that Ms-PE_PGRS38 decreased the expression level of IL-1β (Figure S5D). Our results are aligned with Kim et al.’s findings [27]. However, we investigated different upstream regulators (TLR4/NF-kB), different cell lines (RAW264.7), as well as the LPS pre-treatment strategy for the detection of proinflammatory cytokines. These approaches distinguish our study from that of Kim et al. and contribute to a deeper understanding of the immunological impact of Ms-PE_PGRS38.

### 3.5. PE_PGRS38 Downregulated TLR4/NF-κB Pathway in RAW264.7 Macrophages

**Figure 4:**
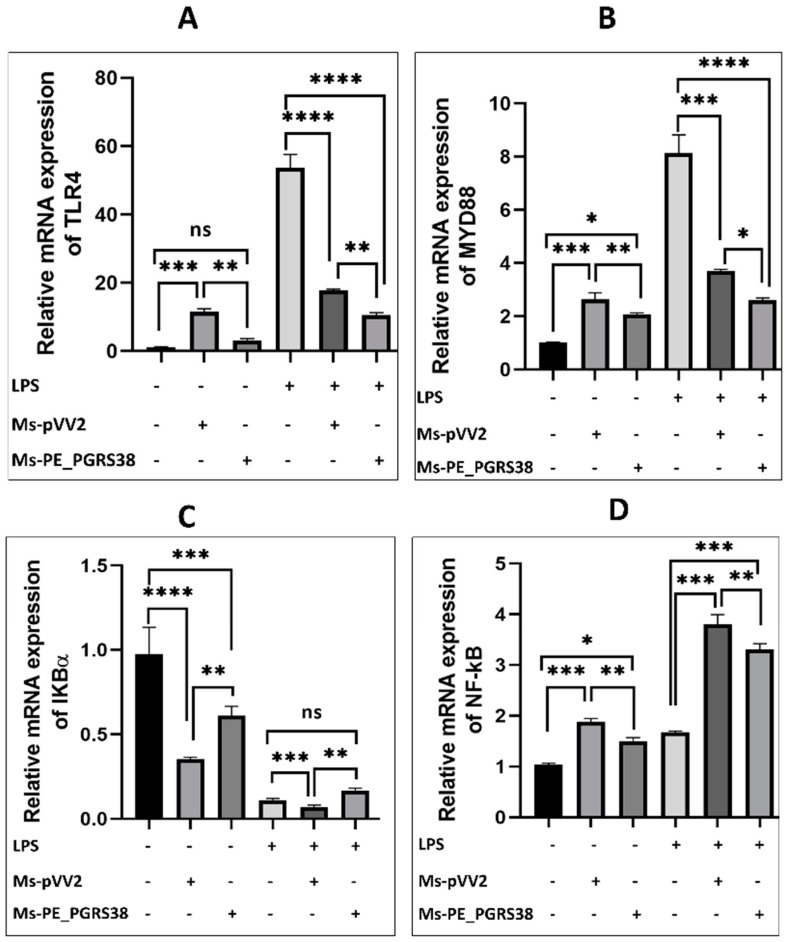
PE_PGRS38 decreased the expression of TLR4/NF-ĸB signaling in infected RAW264.7. Cells were challenged with 0.1 µg/mL LPS for 2 h and subsequently infected with recombinant strains of *M. smegmatis* at 10:1 MOI for 4 h. Total RNA was extracted from cells 24 h post-infection, and mRNA expression of (**A**) TLR4, (**B**) MYD88, (**C**) IKB-α, and (**D**) NF-κB was determined by RT-qPCR assay. ns > 0.05, * *p* < 0.05 ** *p* < 0.01, *** *p* < 0.001, and **** *p* < 0.0001 (one-way ANOVA).

Macrophages are the primary host for Mtb, while the NF-κB pathway is known to induce inflammation during infection [40]. To elucidate the impact of PE_PGRS38 on the NF-κB pathway, RAW264.7 macrophage cells were subjected to infection with recombinant Ms-pVV2 and Ms-PE_PGRS38 strains. RT-qPCR results show that Ms-PE_PGRS38 downregulated the mRNA expression level of TLR4, MYD88, and NF-ĸB while inducing the expression of IKB-α in RAW264.7. Likewise, in LPS-treated cells, Ms-PE_PGRS38 significantly downregulated the mRNA expression of TLR4, MYD88, and NF-ĸB while upregulating the expression of IKB-α (Figure 4). Additionally, immunoblotting analysis implied that Ms-PE_PGRS38 inhibited the expression levels of MYD88, TLR4, p-IKB-α, NF-κB, and p-NF-κB as compared to Ms-pVV2, while Ms-PE_PGRS38 induced the expression level of IKB-α as compared to Ms-pVV2. Similar findings were observed in LPS-treated RAW264.7. Result show that Ms-PE_PGRS38 downregulated the expression of MYD88, TLR4, p-IKB-α, NF-κB, and p-NF-κB as compared to Ms-pVV2, while increasing the expression level of IKB-α *(*Figure S4A–G). These findings indicate that PE_PGRS38 plays a pivotal role in evading the immune responses of the host during infection.

### 3.6. PE_PGRS38 Inhibited NLRP3 Inflammasome in RAW264.7 Macrophages

The involvement of Mtb in the NLRP3-dependent inflammasome during infection is characterized by recent studies [41]. Mycobacterial virulence proteins are either involved in the induction or inhibition of the NLRP3 inflammasome [42,43,44]. We investigated the role of PE_PGRS38 in the NLRP3-dependent inflammasome in RAW264.7 macrophages. Cells were infected with Ms-pVV2 and Ms-PE_PGRS38 strains for 24 h.

**Figure 5:**
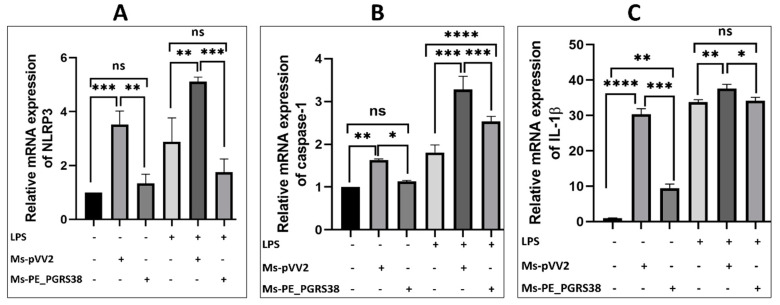
PE_PGRS38 decreased the expression level of NLRP3 inflammasome in infected RAW264.7. Cells were challenged with 0.1 µg/mL LPS for 2 h and subsequently infected with recombinant strains of *M. smegmatis* at 10:1 MOI for 4 h. Total RNA was extracted from cells 24 h post-infection, and mRNA expression of (**A**) NLRP3, (**B**) caspase-1, and (**C**) IL1-β was determined by RT-qPCR assay. ns > 0.05, * *p* < 0.05 ** *p* < 0.01, *** *p* < 0.001, and **** *p* < 0.0001 (one-way ANOVA).

After infection, total RNA was extracted and subjected to RT-qPCR. The results show that Ms-PE_PGRS38 significantly repressed the levels of NLRP3, caspase-1, and IL-1β in infected RAW264.7. Ms-PE_PGRS38 also decreased the expression levels of NLRP3 and IL-1β in LPS-induced RAW264.7 as compared to Ms-pVV2 (Figure 5). Similarly, total proteins were extracted from RAW264.7. The expression level of NLRP3, cleaved caspase-1, and IL-1β were determined through Western blotting. The results show that the expression levels of NLRP3, caspase-1, and IL-1β in RAW264.7 macrophages infected with Ms-PE_PGRS38 were decreased as compared to the Ms-pVV2 strain. Ms-PE_PGRS38 also downregulated the expression of NLRP3, cleaved caspase-1, and IL-1β in LPS-induced RAW264.7 as compared to Ms-pVV2 (Figure S5A–D). Collectively, these results suggest that the PE_PGRS38 protein of Mtb inhibited the NLRP3-dependent inflammasome.

### 3.7. PE_PGRS38 Inhibited Apoptosis of RAW264.7 Macrophages

**Figure 6:**
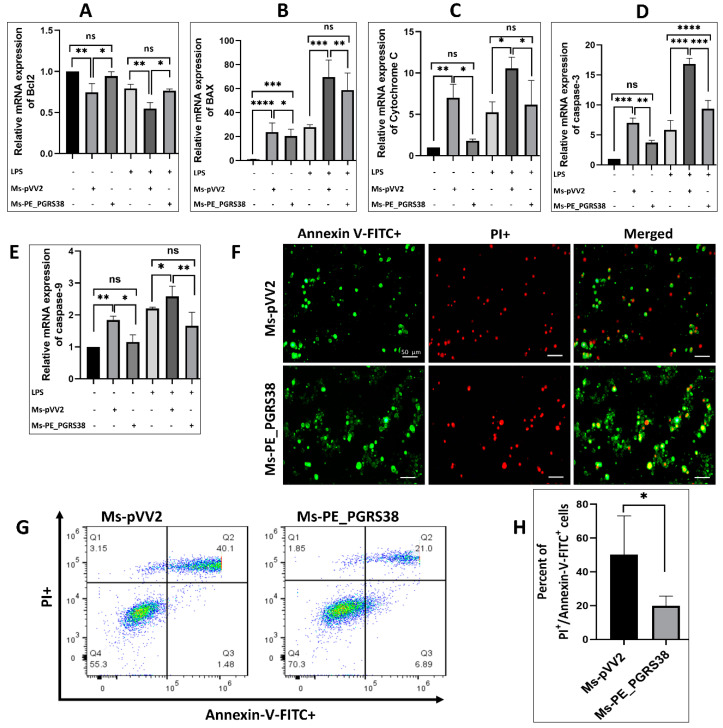
PE_PGRS38 decreased the level of apoptosis in infected RAW264.7. Cells were challenged with 0.1 µg/mL LPS for 2 h and subsequently infected with recombinant strains of *M. smegmatis* at 10:1 MOI for 4 h. Total RNA was extracted from cells 24 h post-infection, and mRNA expression of (**A**) Bcl2, (**B**) Bax, (**C**) cytochrome c, (**D**) caspase-3, and (**E**) caspase-9 was determined by RT-qPCR assay. RAW264.7 cells were collected after 24 h of infection, and the level of apoptosis was determined by fluorescence microscope (**F**) and flow cytometry (**G**,**H**) through annexin-V-FITC and PI straining. Each gate of flow cytometry has four quadrants (Q1–Q4). Q1 represents necrotic cells population, Q2 shows late apoptotic cells, Q3 indicates early apoptotic cells, while Q4 depicts live cell. ns > 0.05, * *p* < 0.05, ** *p* < 0.01 *** *p* < 0.001, and **** *p* < 0.0001 (*t*-test, one-way ANOVA). Scale bar represents 50 µm.

Various studies have highlighted the function of mycobacterial virulence factors in the induction and inhibition of apoptosis during infection [45,46,47]. To study the effect of PE_PGRS38 on apoptosis, RAW264.7 was induced with LPS and subsequently infected with Ms-pVV2 and Ms-PE_PGRS38 for 24 h. RT-qPCR, Western blotting, flow cytometry, and fluorescent microscopy were used to analyze apoptosis. Total RNA from RAW264.7 infected with Ms-pVV2 and Ms-PE_PGRS38 was extracted, and RT-qPCR was carried out. The results show that Ms-PE_PGRS38 decreased the mRNA expression level of Bax, cytochrome c, caspase-3, and caspase-9 as compared to Ms-pVV2. Ms-PE_PGRS38 increased the mRNA expression of Bcl2 as compared to Ms-pVV2. Similarly, in LPS-induced cells, Ms-PE_PGRS38 downregulated the mRNA expression level of Bax, cytochrome c, caspase-3, and caspase-9 and upregulated Bcl2 as compared to Ms-pVV2 (Figure 6A–E). Likewise, Ms-PE_PGRS38 inhibited the expression of proteins associated with apoptosis. Total proteins were isolated from RAW264.7 and infected with Ms-pVV2 and Ms-PE_PGRS38. Through immunoblotting, we found that Ms-PE_PGRS38 inhibited the Bax, cytochrome c, caspase-3, and caspase-9 expression levels in macrophages as compared to Ms-pVV2 while inducing the expression level of Bcl2. Ms-PE_PGRS38 also decreased the Bax, cytochrome c, caspase-3, and caspase-9 expression levels in LPS-treated cells as compared to Ms-pVV2. Ms-PE_PGRS38 increased the expression of Bcl2 protein in LPS-treated RAW264.7 as compared to Ms-pVV2 (Figure S6A,B–F). Flow cytometry is a major technique used to analyze apoptotic cell death. Annexin V-FITC is a marker for early apoptosis. During the early stage of apoptosis, the cell membrane loses integrity, and the membrane phospholipid phosphatidylserine (PS) is translocated from the inner to the outer leaflet of the plasma membrane, thereby exposing PS to the external cellular environment. Annexin V has a high affinity for PS and binds to exposed apoptotic cell surface PS [48], while PI is a nucleic acid-binding dye that binds to DNA during late apoptosis or necrosis. For the measurement of apoptosis levels through immunofluorescence microscopy and flow cytometry, RAW264.7 cells were incubated with annexin V-FITC and PI. The fluorescent microscopy (Figure 6F) and flow cytometry (Figure 6G,H) findings indicated that Ms-PE_PGRS38 substantially inhibited apoptosis (annexin V-FITC+/PI+) of infected RAW264.7 cells as compared to Ms-pVV2. These findings imply that PE_PGRS38 is crucial to *M. smegmatis*’ intracellular survival in RAW264.7 macrophages through inhibition of apoptosis.

### 3.8. Effect of PE_PGRS38 on Lungs’ Hisopathology

A previous study reported that PE_PGRS38 decreased the pathogenesis of the lungs during infection [27]. In this study, tissue sections of infected mice were stained with H&E and observed under a compound microscope. In line with the previous study, our results show that both Ms-pVV2 and Ms-PE_PGRS38 caused the infiltration of inflammatory cells into the infectious region after 4 days of infection. However, the amount of acute inflammatory cells in the lungs infected with Ms-pVV2 was significantly higher than Ms-PE_PGRS38. Emphysema was prominent in both Ms-pVV2- and Ms-PE_PGRS38-infected lungs 4 days post-infection. After 8 days of infection, septal thickness and erythema were increased in Ms-pVV2-infected lungs as compared to Ms-PE_PGRS38 (Figure S7).

### 3.9. PE_PGRS38 Downregulated the TLR4/NF-κB Signaling In Vivo

**Figure 7:**
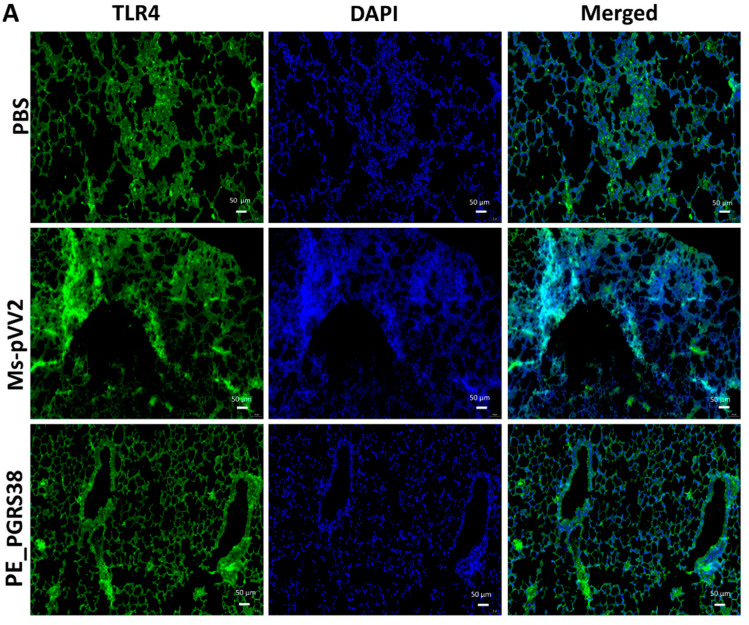

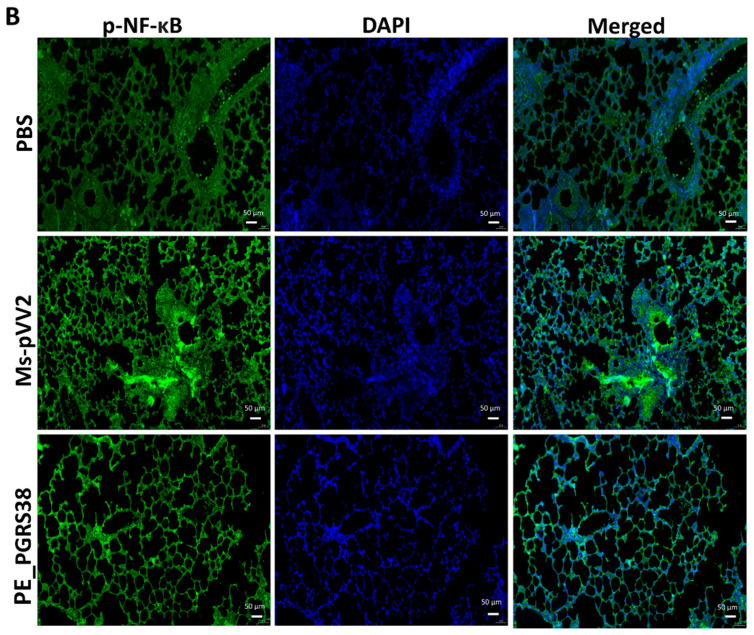
Detection of inflammation through immunofluorescence assay for recombinant *M. smegmatis*-infected lungs of mice. Mice were infected, as aforementioned. (**A**) represents the expression of TLR4, and (**B**) indicates the level of p-NF-κB expression in the lung tissues. The scale bar represents 50 µm.

NF-κB regulates several biological functions, including immunological and inflammatory responses, cell growth, and apoptosis, by inducing various target genes. The RT-qPCR and immunoblot results show that PE_PGRS38 downregulated TLR4/NF-κB signaling in RAW264.7 cells (Figure 4 and Figure S4). We performed an immunofluorescent assay after infecting mice with recombinant Ms-pVV2 and Ms-PE_PGRS38 to validate the expression levels of TLR4 and p-NF-B in vivo. The results show that Ms-PE_PGRS38 decreased TLR4 expression in infected lungs as compared to the Ms-pVV2 strain (Figure 7A). Likewise, Ms-PE_PGRS338 decreased p-NF-κB expression in infected lungs compared with Ms-pVV2 (Figure 7B). The in vivo immunofluorescent findings show consistency with RT-qPCR and immunoblot results, which indicates that PE_PGRS38 downregulates the expression of TLR4 and NF-κB in the host during infection.

## 4. Discussion

Members of the *Mycobacterium* complex possess a unique cell-wall-associated PE_PGRS protein family that consists of 99 members and covers 4% of the Mtb genome [5]. Mycobacteria acquire new pathogenic features due to PE_PGRS genes [49]. PE_PGRS proteins cause the most antigenic variation, decrease cytokine production [23], and impede macrophage phagolysosome maturation [50]. Despite intense research, the significance of the majority of PE_PGRS proteins in Mtb pathophysiology and biology is yet to be explored. PE_PGRS38, encoded by Rv2162c in Mtb, and its orthologs are present in members of the *Mycobacterium* complex, such as *M. marinum* [6].

In this study, we engineered a recombinant *M. smegmatis* (Ms-PE_PGRS38) that expresses Mtb PE_PGRS38 in *M. smegmatis* (Figure S1A–C). We found that PE_PGRS38 is expressed in the cell wall of *M. smegmatis* (Figure S1D). Though Ms-pVV2 and Ms-PE_PGRS38 have similar growth kinetics (Figure S1E), the enhanced biofilm formation of Ms-PE_PGRS38 could be due to the influence of PE_PGRS38 on the cell wall of *M. smegmatis* (Figure 1). For successful elimination inside the host cell, mycobacterium is exposed to reactive oxygen species such as H_2_O_2_ and acidic pH in the phagosome during phagocytosis [51]. By exposing the recombinant strains to exogenous H_2_O_2_, acidic pH, and SDS stress, we found that PE_PGRS38 of Mtb promoted the viability of recombinant *M. smegmatis* under H_2_O_2_ stress (Figure 2A). Under acidic pH and SDS stress conditions, we found that the growth rate of recombinant Ms-PE_PGRS38 was extensively reduced as compared to Ms-pVV2 (Figure 2B,C). The sensitivity of recombinant *M. smegmatis* could be an outcome of the permeability of the cell wall, caused by PE_PGRS38 expression.

Mtb effectors, especially various PE_PGRS family proteins, increase mycobacterial viability inside macrophages, either by inducing [52] or inhibiting the production of proinflammatory cytokines [27,53,54]. A recent study reported that PE_PGRS38 enhanced the survival of *M. smegmatis* in BMDM and 293T cells and in the lungs of mice [27]. In line with the previous investigation on PE_PGRS38, our results show that PE_PGRS38 enhanced the cellular survival of recombinant *M. smegmatis* in RAW264.7 macrophages and in the lungs of infected mice (Figure S2A,B). Resistance to H_2_O_2_ stress could be a contributing factor for intracellular survival. As Ms-pVV2 and Ms-PE_PGRS38 possessed similar growth properties (Figure S1E), the higher level of survival of Ms-PE_PGRS38 in macrophages could be attributed to the impact of PE_PGRS38 on innate immunity.

Mtb has sophisticated immune evasion strategies to redirect or disrupt host proteins that neutralize the pathogenicity of pathogens. Several PE_PGRS proteins are linked with the immune evasion strategy of the mycobacteria by inducing proinflammatory signals [16,39,55]. Various studies have also found that PE_PGRS proteins inhibit proinflammatory responses [56]. Recently, Kim et al. reported that PE_PGRS38 decreased the expression of TRAF6-dependent proinflammatory responses including TNF-α, IL-6, IL-10, and IL-1β in BMDM and 293T cell lines [27]. In agreement with Kim et al.’s findings, our results show that PE_PGRS38 inhibits proinflammatory signals including IL-1β, TNF-α, and IL-6 while inducing the expression of IL-10 in LPS-primed RAW264.7 (Figure 3, Figure 5C and Figure S3A–D). The increased level of IL-10 caused impaired phagosome maturation and inflammatory markers in infected macrophages, as described by previous studies [57,58].

During mycobacterial infection, macrophages are the primary host for Mtb [40]. NF-κB is a transcriptional regulator that influences immune activation and apoptosis by regulating cytokine production during infection [59]. As PE_PGRS of *M. tuberculosis* are genetically varied [60], therefore, the anti-mycobacterial immune responses of the host are different. PE_PGRS11, PE_PGRS30, and the PGRS sequence of PE_PGRS30 are reported to be key mediators of TLR/NF-κB-dependent inflammation [22,61]. However, PE_PGRS31 is involved in the inhibition of NF-κB signaling [62]. In this study, normal or LPS-treated RAW264.7 macrophages were processed for infection with recombinant *M. smegmatis* strains. Our results show that PE_PGRS38 substantially downregulated TLR4, p-IKB-α, Myd88, p-NF-κB, and NF-κB in normal and LPS-treated RAW26.7 macrophages during infection, while expression of IKB-α upregulated, as shown in Figure 4 and Figure S4. Furthermore, the immunofluorescence results of the lungs of mice show that PE_PGRS38 downregulated the expression of TLR4 and p-NF-κB during infection (Figure 7A,B). Our in vitro and in vivo findings related to inflammation demonstrate that PE_PGRS38 evaded the host anti-mycobacterial defense mechanisms by downregulating TLR4/NF-κB-dependent inflammatory responses.

Priming with microbial elements, such as TLR ligands, or endogenous compounds, such as tumor necrosis factor, triggers NLRP3 expression through NF-κB activation [63]. The role of Mtb in the NLRP3-dependent inflammasome is characterized by recent studies [64]. In this study, we investigated that PE_PGRS38 inhibited the expression of NLRP3, caspase-1, and IL-1β in both LPS-induced and uninduced RAW264.7, as shown in Figure 5 and Figure S5. As aforementioned, PE_PGRS38 decreased the expression of NF-κB; therefore, downregulation of NLRP3, caspase-1, and IL-1β could be the downstream outcome of the TLR4/NF-κB signaling cascade.

The Mtb exploits host defenses to survive. Apoptosis prevents intracellular pathogens like Mtb from releasing and spreading mycobacterial infection; however, it damages tissue [65]. Mycobacterial PE_PGRS are virulence factors and have roles in the host’s cell apoptosis [66]. Among these, PE_PGRS33 and PE_PGRS5 are known to mediate apoptotic cell death [17,67]. However, PE_PGRS18, PE_PGRS41, and PE_PGRS62 are involved in the inhibition of apoptosis [19,23,45]. In this study, we found that PE_PGRS38 inhibits apoptosis in RAW264.7 cells. PE_PGRS38 downregulated the expression of mitochondrial apoptotic effector cytochrome c. PE_PGRS38 also decreased the expression of Bax, cleaved caspase-3, and cleaved caspase-9 in RAW264.7 cells while increasing the level of Bcl-2 expression (Figure 6A–E and Figure S6A–F). In addition, results of florescent microscopy and flow cytometry show that PE_PGRS38 decreased the apoptosis level in infected RAW264.7 cells (Figure 6F–H). These results reveal that PE_PGRS38 increased the intracellular burden by inhibiting apoptotic markers.

## 5. Conclusions

In a nutshell, this study demonstrates that PE_PGRS38 is expressed in the cell wall and contributes to cell permeability and biofilm formation. PE_PGRS38 inhibited the antimycobacterial immune responses by downregulating TLR4/NF-κB-dependent inflammation apoptosis and the NLRP3 inflammasome. PE_PGRS38 enabled recombinant *M. smegmatis* to resist killing and enhance colonization within the host. Revelations of these attributes of PE_PGRS38 highlight its significance in Mtb virulence and host–pathogen interaction and make it an important target for novel therapeutic interventions against tuberculosis, thus, fulfilling the primary aim of this study. Further research would offer a better comprehension of the mechanisms employed by PE_PGRS38 to enable the intracellular lifestyle, modulation of immune responses, and enhanced pathogenicity of Mtb.

## Data Availability

The original data for this work are available upon email request to the corresponding author.

## Supplementary Materials

The following supporting information can be downloaded at: https://www.mdpi.com/article/10.3390/biology13050313/s1.
