# Supplementary material for: Mycobacterium tuberculosis PE_PGRS38 Enhances Intracellular Survival of Mycobacteria by Inhibiting TLR4/NF-κB-Dependent Inflammation and Apoptosis of the Host
Source: Biology (Basel). 2024 Apr 30;13(5):313. doi: 10.3390/biology13050313 (PMC11118070; doi:10.3390/biology13050313)
Supplement: Supplementary file 1 [file biology-13-00313-s001.zip › biology-2905602-supplementary.pdf]

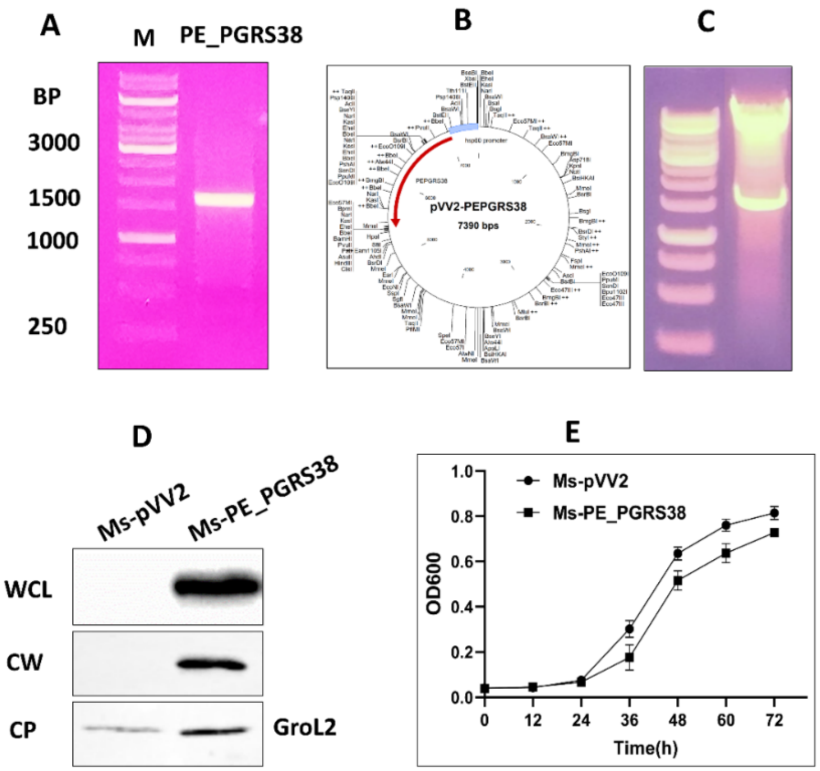

Figure S1: Confirmation, subcellular localization of PE_PGRS38, and growth of recombinant strains (Ms-pVV2 and Ms-PE_PGRS38) of *M. smegmatis* mc^{2}155. **(A)** shows the PCR product of PE_PGRS38, **(B)** represents the virtual construct, **(C)** shows the digested form of expression vector pVV2-PE_PGRS38 **(D)** Fractions of recombinant *M. smegmatis* strains were subjected to Western blot to determine the subcellular localization of PE_PGRS38. GroL2 (56 kDa) is expressed in the cytoplasm of *M. smegmatis.* GroL2 serves as a control; WCL represents the whole cell lysate; CW depicts the cell wall; CP indicates the cytoplasm. **(E)** Ms-pVV2 and Ms-PE_PGRS38 were maintained in 7H9 enhanced with Kan (25 µg/ml), Glycerol 0.2%, and Tween 80 (0.05%). The OD_{600} was calculated at 12-hour intervals.

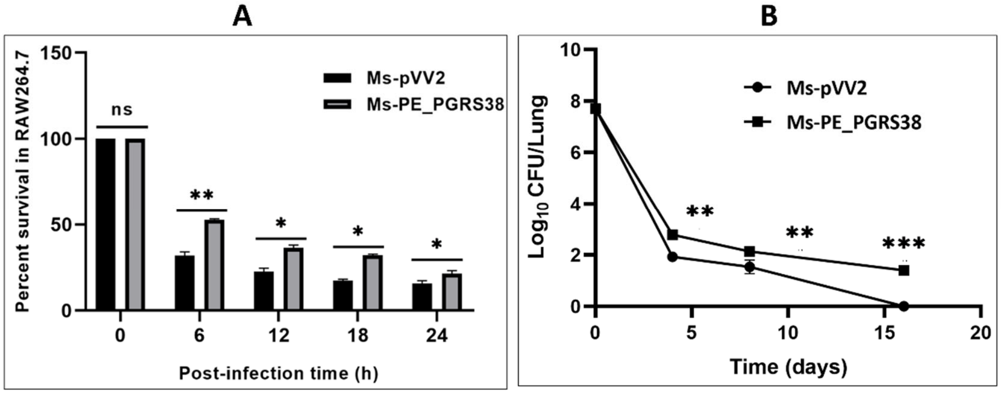

Figure S2: PE_PGRS38 enhanced the intracellular survivability of *M. smegmatis* in infected RAW264.7 macrophage cells and C57BL/6J mice. RAW264.7 and C57BL/6J mice were infected with Ms-pVV2 and Ms-PE_PGRS38. Cells and lungs tissues were washed with PBS at indicated timepoints post-infection, lysed, and plated in 7H11 medium to determine the number of recombinant *M. smegmatis* strains. NS: not significant. NS > 0.05, **p* < 0.05, ***p* < 0.01, ****p* < 0.001 (student t-test).

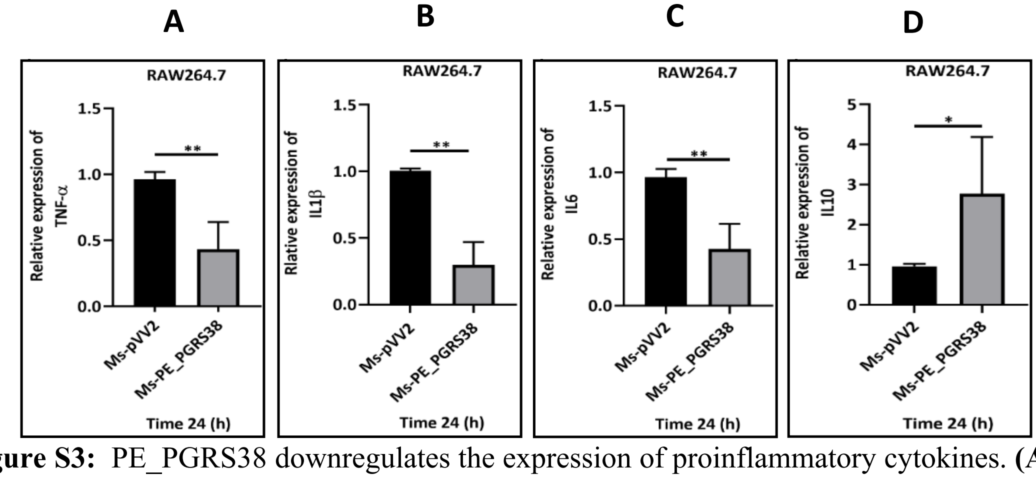

Figure S3: PE_PGRS38 downregulates the expression of proinflammatory cytokines. **(A–D)** showing the relative mRNA expression of TNF-α, IL-1β, IL-6, and IL10 in RAW264.7 infected with recombinant strains of *M. smegmatis.* **P* < 0.05 and ***P* < 0.01 (t-test).

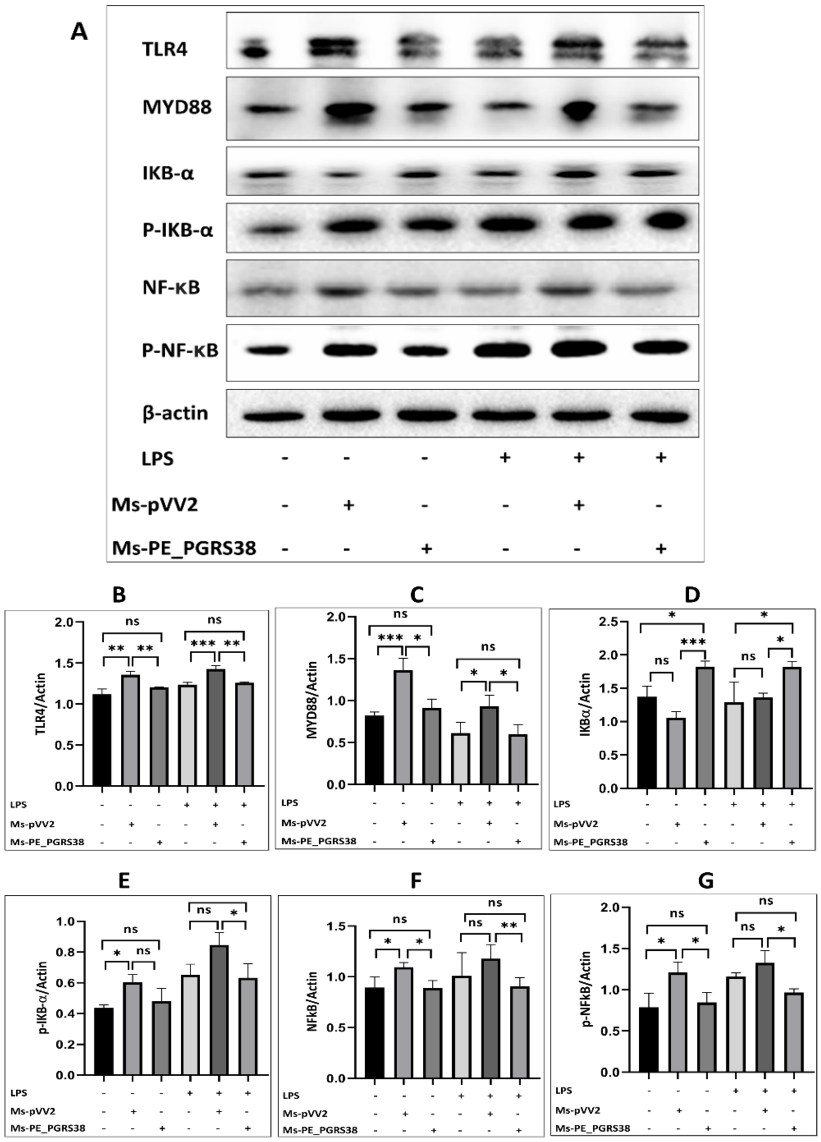

Figure S4:

PE_PGRS38 downregulates NF-κB signaling in infected RAW264.7. Cells were challenged with 0.1 µg/ml LPS for 2 hours and subsequently infected with recombinant strains of *M. smegmatis* at 10:1 MOI for 4 hours. **(A)** Proteins were extracted from cells 24 hours post-infection, and expression of TLR4, MYD88, IKB-α, p-IKBα, NF-κB, p-NF-κB, and total β-actin was determined by western blot-assay. **(B-G)** Represents the densitometric analysis of

TLR4, MYD88, IKB-α, p-IKBα, NF-κB, and p-NF-Kb, respectively. NS > 0.05, *$p < 0.05$, and **$p < 0.01$ (One-way ANOVA).

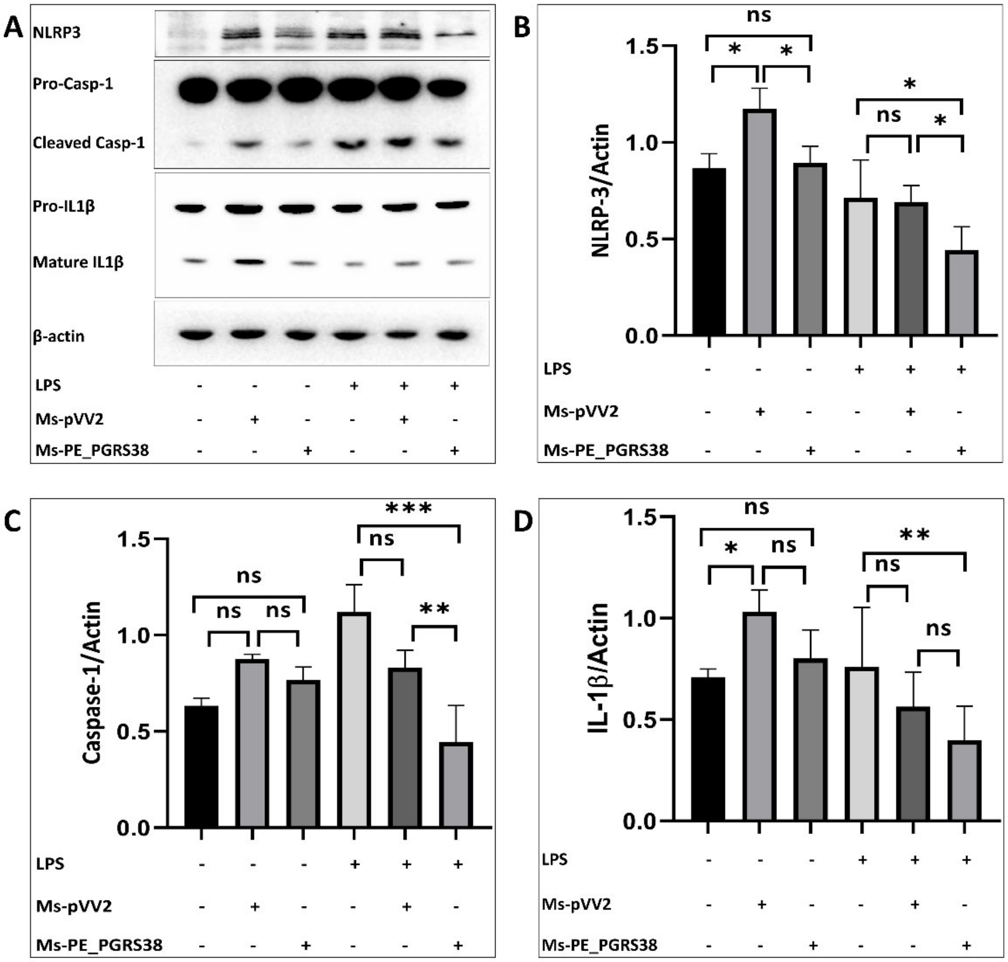

Figure S5: PE_PGRS38 protein decreased the NLRP3 inflammasome. RAW264.7 cells were challenged with 0.1 µg/ml LPS for 2 hours and subjected to infection with recombinant strains of *M. smegmatis* for 4 hours. **(A)** Proteins were extracted from cells 24 hours post-infection, and the expression levels of NLRP3, Caspase-1, and IL1β and total β-actin were determined by western blot assay. **(B-D)** Represents the densitometric analysis of NLRP3, caspase-1, and IL1β. NS > 0.05, *$p < 0.05$, and **$p < 0.01$ (one-way ANOVA).

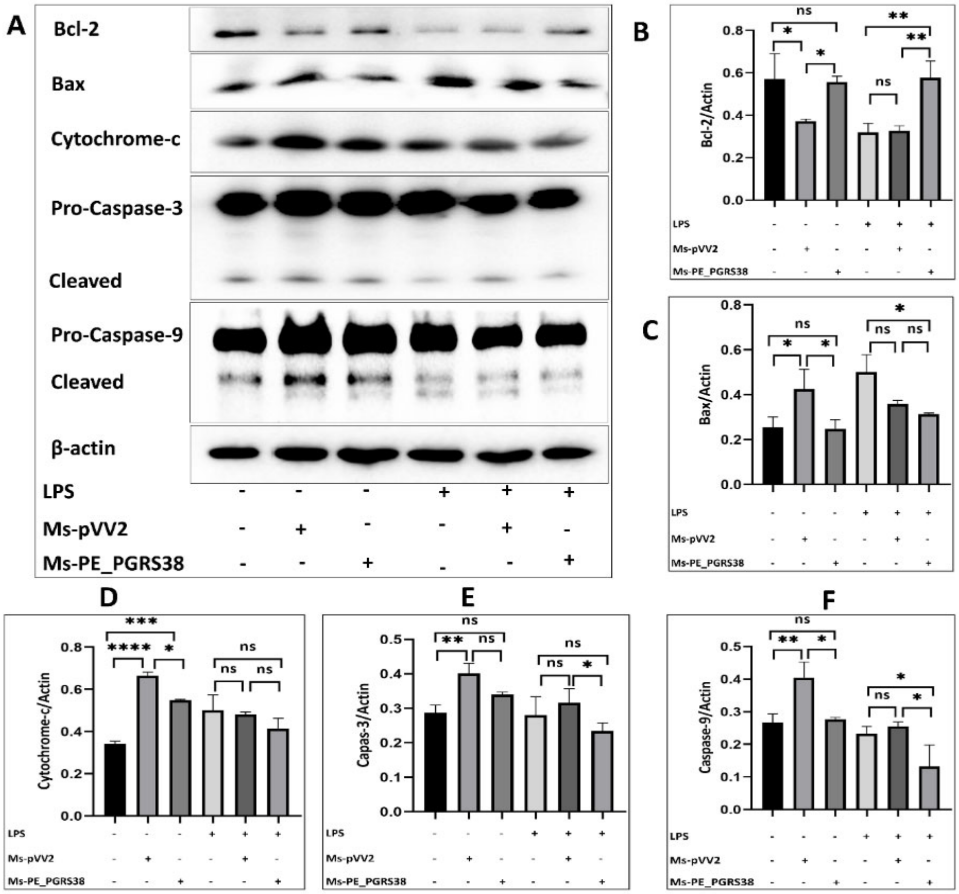

Figure S6: PE_PGRS38 decreased the expression level of apoptosis in infected RAW264.7. Cells were challenged with 0.1 μg/ml LPS for 2 hours and subsequently infected with recombinant strains of *M. smegmatis* at 10:1 MOI for 4 hours. Proteins were extracted from cells 24 hours post-infection, and the expression levels of Bax, Bcl-2, cytochrome-c, caspase-3, caspase-9, and total β-actin were determined by Western blot assay. **(A)** represents blots, while **(B-F)** depicts the densitometric analysis of Bcl2, Bax, cytochrome-c, caspase-3, and caspase-9. NS > 0.05, **p* < 0.05 ***p* < 0.01, ****p* < 0.001, and *****p* < 0.0001 (One-way ANOVA).

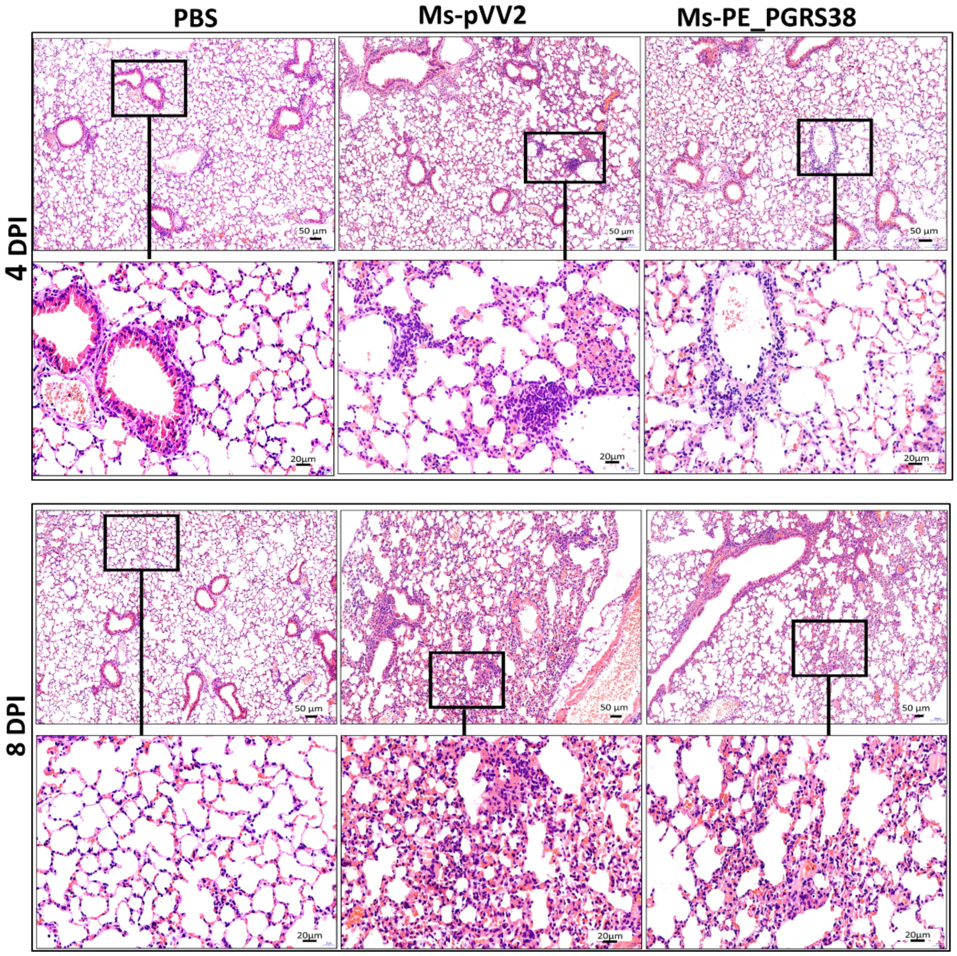

Figure S7: Lung histology of infected mice with Ms-pVV2 and Ms-PE_PGRS38 strains. C57BL/6J were subjected to aerosolization, as discussed previously. The left lobe of each lung was fixed with 4% PFA for pathological analysis. The Ms-pVV2 or Ms-PE_PGRS38-infected lung sections were organized on 4 and 8 days post-infection (DPI) and stained with H&E. The tissue sections were visualized using 40X microscope objectives. Scale bars = 50 and 20 µm.

Table S1: List of the primer sequence used in this study.

Gene	5'-3' Forward primer	5'-3' Reverse primer
PE_PGRS38	GG<u>CATATG</u>TCGTTTGTGATTGCGGCG	AT<u>GGATCC</u>TCAGCCGTCGGCGCCGTTTG
TNF-α	ACTGAACTTCGGGGTGATCGGT	TGGTTTGCTACGACGTGGGCTA
IL-1β	TGCCACCTTTTGACAGTGATGAG	TGATGTGCTGCTGCGAGATTT
IL-6	CCCCAATTTCCAATGCTCTCC	CGCACTAGGTTTGCCGAGTA
IL-10	TGAGGCGCTGTCGTCATCGATTTCTCCC	ACCTGCTCCACTGCCTTGCT
Bax	AGGATGCGTCCACCAAGAAGCT	TCCGTGTCCACGTCAGCAATCA
Bcl2	CCTGTGGATGACTGAGTACCTG	AGCCAGGAGAAATCAAACAGAGG
Cyto-c	GAGGCAAGCATAAGACTGGACC	ACTCCATCAGGGTATCCTCTCC
Caspase-3	GGAGTCTGACTGGAAAGCCGAA	CTTCTGGCAAGCCATCTCCTCA
Caspase-9	GCTGTGTCAAGTTTGCCTACCC	CCAGAATGCCATCCAAGGTCTC
NLRP3	TCACAACTCGCCCAAGGAGGAA	AAGAGACCACGGCAGAAGCTAG
Caspase-1	GGCACATTTCCAGGACTGACTG	GCAAGACGTGTACGAGTGGTTG
NF-κB	TCCTGTTCGAGTCTCCATGCAG	GGTCTCATAGGTCCTTTTGCGC
IKB-α	GCCAGGAATTGCTGAGGCACTT	GTCTGCGTCAAGACTGCTACAC
TLR4	AGCTTCTCCAATTTTTCAGAACTTC	TGAGAGGTGGTGTAAGCCATGC
MYD88	ACCTGTGTCTGGTCCATTGCCA	GCTGAGTGCAAACTTGGTCTGG
β-actin	TGACGTTGACATCCGTAAAGACC	CTCAGGAGGAGCAATGATCTTGA
